# Age and sex differences in outpatient antipsychotic prescriptions for schizophrenia: a claims data study

**DOI:** 10.1007/s00406-024-01867-z

**Published:** 2024-09-30

**Authors:** Tabea Ramin, Jens-Uwe Peter, Michael Schneider, Martin Heinze, Oliver Riedel, Sophie Hanna Langbein, Ulrike Haug, Oliver Zolk

**Affiliations:** 1 Institute for Clinical Pharmacology, Brandenburg Medical School, Immanuel Klinik Rüdersdorf, Seebad 82/83, 15562 Rüdersdorf, Germany; 2 Faculty of Health Sciences, Joint Faculty of the University of Potsdam, The Brandenburg Medical School Theodor Fontane and the Brandenburg University of Technology Cottbus–Senftenberg, Karl-Liebknecht-Str. 24-25, 14476 Potsdam, Germany; 3 Department for Psychiatry and Psychotherapy, Brandenburg Medical School, Immanuel Klinik Rüdersdorf, Seebad 82/83, 15562 Rüdersdorf, Germany; 4 Department of Clinical Epidemiology, Leibniz Institute for Prevention Research and Epidemiology–BIPS, Achterstraße 30, 28359 Bremen, Germany; 5 Department of Biometry and Data Management, Leibniz Institute for Prevention Research and Epidemiology–BIPS, Achterstraße 30, 28359 Bremen, Germany; 6 Faculty of Human and Health Sciences, University of Bremen, Bremen, Germany

**Keywords:** Antipsychotic drugs, Schizophrenia, Geriatric psychiatry, Elderly

## Abstract

Most studies on antipsychotic efficacy and safety, including sex differences, focus on young schizophrenia patients. However, with an aging population, the number of older schizophrenia patients is increasing. This group faces challenges due to varying treatment responses and higher risks of adverse reactions, and guidelines often lack specific recommendations due to insufficient trials. Therefore, we investigated how age and sex influence antipsychotic prescribing practices in schizophrenia using the German Pharmacoepidemiological Research Database (GePaRD). We included patients diagnosed with schizophrenia (ICD-10 code F20.X) who had been prescribed at least one antipsychotic on an outpatient basis in at least two consecutive quarters in 2020, analyzing prescription data for 49,681 patients. Key findings include a notable preference for second-generation antipsychotics (SGAs) across all age groups, especially in younger patients, possibly due to their perceived better tolerability and efficacy. Treatment intensity with SGAs (expressed as the defined daily doses of SGAs per patient in 2020) initially increased with age, peaked among 35- to 44-year-olds, and then decreased, with the lowest treatment intensity in patients aged 65 years and older. The prescription patterns of specific SGAs and first-generation antipsychotics varied across age groups, highlighting the complexity of treatment decisions in schizophrenia management. Sex differences in prescription frequency and treatment intensity were also observed. The basic recommendation of the guideline to consider sex and age when prescribing antipsychotics therefore appears to be followed. Whether this prescribing practice is really optimal for older male and female schizophrenia patients, however, still needs to be proven in clinical trials.

**Supplementary Information:**

The online version contains supplementary material available at 10.1007/s00406-024-01867-z.

## Introduction

The global population is currently experiencing a significant aging trend, with projections indicating that the proportion of older individuals in the total population will almost double from 12% in 2015 to around 22% by 2050 [1]. According to the World Health Organization (WHO), 20% of individuals over the age of 60 grapple with some form of mental or neurological disorder, emphasizing the critical need to prioritize the treatment and management of mental illness in the elderly population [2].

While the onset of schizophrenia typically occurs between the ages of 15 and 35, with approximately 65% of cases emerging before the age of 30, individuals aged 65 years and older are expected to constitute over a quarter of the total population with schizophrenia due to changing demographics [3]. When discussing schizophrenia in the elderly, terms such as "geriatric schizophrenia", "later-life schizophrenia", or "late-onset schizophrenia" are employed, reflecting different age groups. Conventionally, the geriatric population includes individuals aged 65 years and older, a category that also encompasses geriatric schizophrenia patients. Later-life schizophrenia consists of two distinct groups: those individuals diagnosed with schizophrenia early in life (late adolescence or young adulthood) who are now middle-aged or older, and those first diagnosed when they are older than the typical age of onset, i.e., 40–45 years or older, also known as late-onset schizophrenia [2].

Controversy exists regarding whether symptoms of schizophrenia may differ between younger and older patients with schizophrenia, particularly in relation to the variation of positive and negative symptoms with age of onset [4–6]. Vahia et al. found that positive symptoms and general psychopathology were less severe in late-onset schizophrenia (LOS) patients than in early-onset (EOS) patients, but the groups did not differ in the severity of negative symptoms [6]. While the study by Mason et al. on 269 schizophrenia patients found that similarities between EOS and LOS far outweighed the differences across a range of symptoms and measures. The LOS and EOS groups performed similarly on the Positive and Negative Syndrome Scale (PANSS) positive, negative, global, and overall totals [5]. Regardless, the use of antipsychotic medications in the treatment of this condition is recommended for all age groups [3].

In addition to age, sex also has an influence on the course of schizophrenia, particularly with regard to age at onset, symptoms, and responses to treatment. While the lifetime prevalence is similar in men and women, schizophrenia is more prevalent among men under 40, with peak incidence between 20 and 25 years. In women, higher prevalence is found after 40, with two incidence peaks: 20–39 years and during perimenopause [7, 8]. Regarding sex differences in disease presentation men with schizophrenia often have a higher risk of prominent negative symptoms, while women more frequently exhibit affective symptoms [9–11]. Women also tend to have a better response to antipsychotic medication, with larger mean symptom reductions compared to men. The number needed to treat for a response in women is 6.9, compared to 9.4 in men [12]. Several factors contribute to these differences. For example, the better treatment adherence among female patients may play a role [13]. Variations in cytochrome P450 (CYP) enzymes that metabolize antipsychotics may also explain why the optimal dose differs between men and women. Because CYP activity is influenced by gonadal hormone levels, meaning women's reproductive state or exogenous hormone intake can affect dose requirements [13]. Generally, the male-based dose recommendations in the SmPC are too high for women, leading to unnecessarily severe side effects. Twice as many women as men report severe side effects from antipsychotics [14, 15].

The drug treatment of schizophrenia in the elderly poses unique challenges, as this population is particularly vulnerable to the side effects associated with antipsychotics. These side effects often include anticholinergic, metabolic, motor, and cardiac complications [16–18]. Tardive dyskinesias represent a challenging spectrum of side effects that can significantly impair patients' quality of life. For instance, the use of first-generation neuroleptics in elderly patients is associated with an incidence of tardive dyskinesia exceeding 20% in the first year of treatment, increasing to over 50% within three years of cumulative treatment [2, 19, 20]. Multimorbidity, concurrent medication usage, and reduced physiological reserves contribute to an elevated risk of adverse drug reactions and drug-drug interactions. Changes in drug kinetics, receptor sensitivity, and potentially altered responses of positive and negative symptoms to antipsychotic treatment further complicate determining the optimal effective exposition of antipsychotics with advancing age [21–23].

Although considering the safety and efficacy of antipsychotic medications in older patients with schizophrenia is crucial, limited information is available regarding patterns of use in older adults. In this cross-sectional study, we utilize routine health insurance claims data to assess antipsychotic drug (APD) prescribing patterns in patients with schizophrenia aged ≥ 65 years compared with younger patient groups. We investigated the utilization of major APD classes and individual APDs by age and sex. Utilizing a large database comprising approximately 20% of all insured patients in Germany, this study extends the results of our previous study, which focused less representatively on inpatients from a limited number of psychiatric hospitals [24].

## Methods

### Data source

The present analyses utilized data from the German Pharmacoepidemiological Research Database (GePaRD). GePaRD contains claims data from four statutory health insurance (SHI) providers in Germany, encompassing information on approximately 25 million individuals insured with one of the participating providers since 2004 or later. Besides demographic data, GePaRD contains information on medications administered on an outpatient basis. Diagnoses may have been made either on an outpatient basis (by general practitioners and specialists) or during inpatient care. Per data year, there is information on approximately 20% of the general population and all geographical regions of Germany are represented [25].

### Study population and design

In this cross-sectional study, analyses were conducted based on claims data from the year 2020, encompassing all SHI members with continuous coverage from January 1st to December 31st (or until death). A maximum insurance gap of 30 days was permitted. From this population, individuals meeting the following inclusion criteria were selected for analysis:

1. At least one inpatient diagnosis for schizophrenia (ICD-10 code F20.X) and at least one dispensation for antipsychotic medication (ATC code: N05A) in each of at least two consecutive quarters.

or

2) At least two outpatient confirmed diagnoses for schizophrenia (ICD-10 code F20.X) and at least one dispensation for antipsychotic medication (ATC code: N05A) in each of at least two consecutive quarters.

Data on age, sex, APD prescriptions, and co-medication were extracted from the database.

### Classification of dispensed psychoactive drugs

Psychoactive drugs were categorized according to Anatomical Therapeutic Chemical (ATC) codes as follows: psycholeptics (N05), APDs (N05A), anxiolytics (N05B), lithium salts (N05AN), psychoanaleptics (N06), antidepressant drugs (N06A), anticonvulsant drugs (N03), antiparkinsonian drugs (N04), and other nervous system drugs (N07). APDs were further classified into high-potency and low-potency first-generation antipsychotic drugs (FGAs) and second-generation antipsychotic drugs (SGAs).

### Data analysis

To determine the utilization of specific APDs, prescription frequencies were calculated, representing the proportion of patients with at least one prescription of the respective APD dispensed in 2020. Prescription frequencies were assessed for individual APDs or APD classes in the total study population or in respective age or sex subgroups. Age groups were defined as follows (in years): < 25, 25–34, 35–44, 45–54, 55–64, 65–74, and ≥ 75.

Treatment intensity of a given APD per patient during the one-year study period was determined by calculating the dispensed defined daily doses (DDDs) based on prescriptions per patient, expressed as median and interquartile range. The total APD exposure per patient was computed by summing up the DDDs of all prescribed APDs for each patient. The 2023 DDD definition of the World Health Organization [1] was employed for this calculation, using the formula: DDDs/patient/year = (N × M × Q)/(DDD × T), where N is the number of prescriptions dispensed, M is the dose in milligrams or grams, Q is the pack size, DDD is the figure assigned in the WHO DDD definition list, and T is the number of years of the study duration (i.e., 1 year). Treatment intensity of APDs was stratified by sex and age groups.

Depending on data levels, summary statistics comprised counts, percentages, means and standard deviations (SD), medians and interquartile range (IQR), where appropriate. All statistical analyses were conducted using SAS 9.4.

## Results

### Study population

The study included 49,681 health-insured individuals with a primary diagnosis of schizophrenia who met the inclusion criteria. Their mean age was 53.2 ± 16.1 years. Men had a lower mean age of 48.5 ± 15.0 years compared to women (57.6 ± 15.8 years). Patients aged 55 to 64 years formed the largest group (23.6%), with 13.2% and 10.7% belonging to the age groups 65 to 74 and ≥ 75 years, respectively. Overall, more women than men were included (52.5% versus 47.5%). The proportion of women increased with age, ranging from 31.3% in the 25–34 age group to 76.7% in the ≥ 75 years age group (Table 1). Paranoid schizophrenia (F20.0) was diagnosed in 59.7% of patients (62.0% men, 57.6% women) and, along with non-specific schizophrenia (F20.9), was the most frequently diagnosed type of schizophrenia (22.4%). The frequency of other types ranged from 0.8% to 8.1% (Table 1).

**Table 1:** Demographic and clinical characteristics of the study population

Patients included in the analysis (n)	All patients (49,681)	Men (23,619)	Women (26,062)
Primary diagnosis; n (%)			
F20.0 paranoid subtype	29,663 (59.7)	14,643 (62.0)	15,020 (57.6)
F20.1 disorganized (hebephrenic) subtype	869 (1.7)	510 (2.2)	359 (1.4)
F20.2 catatonic subtype	467 (0.9)	222 (0.9)	245 (0.9)
F20.3 undifferentiated subtype	763 (1.5)	369 (1.6)	394 (1.5)
F20.4 postschizophrenic depression	563 (1.1)	244 (1.0)	319 (1.2)
F20.5 residual subtype	4043 (8.1)	1808 (7.7)	2235 (8.6)
F20.6 simple-type schizophrenia	317 (0.6)	173 (0.7)	144 (0.6)
F20.8 other schizophrenia	1885 (3.8)	750 (3.2)	1135 (4.4)
F20.9 schizophrenia, unspecified	11,111 (22.4)	4900 (20.7)	6211 (23.8)
Age years			
Mean (SD)	53.2 (16.1)	48.3 (15.0)	57.6 (15.8)
Median (Q1; Q3)	54 (41; 64)	48 (37; 59)	58 (47; 68)
Age groups; n (%)			
< 25 years	1549 (3.1)	1015 (4.3)	534 (2.0) 1
25–34 years	5209 (10.5)	3576 (15.1)	1633 (6.3)
35–44 years	9034 (18.2)	5814 (24.6)	3220 (12.4)
45–54 years	10,282 (20.7)	5001 (21.2)	5281 (20.3)
55–64 years	11,722 (23.6)	4775 (20.2)	6947 (26.7)
65–74 years	6552 (13.2)	2195 (9.3)	4357 (16.7)
≥ 75 years	5333 (10.7)	1243 (5.3)	4090 (15.7)

### Use of specific APDs

**Fig. 1:**
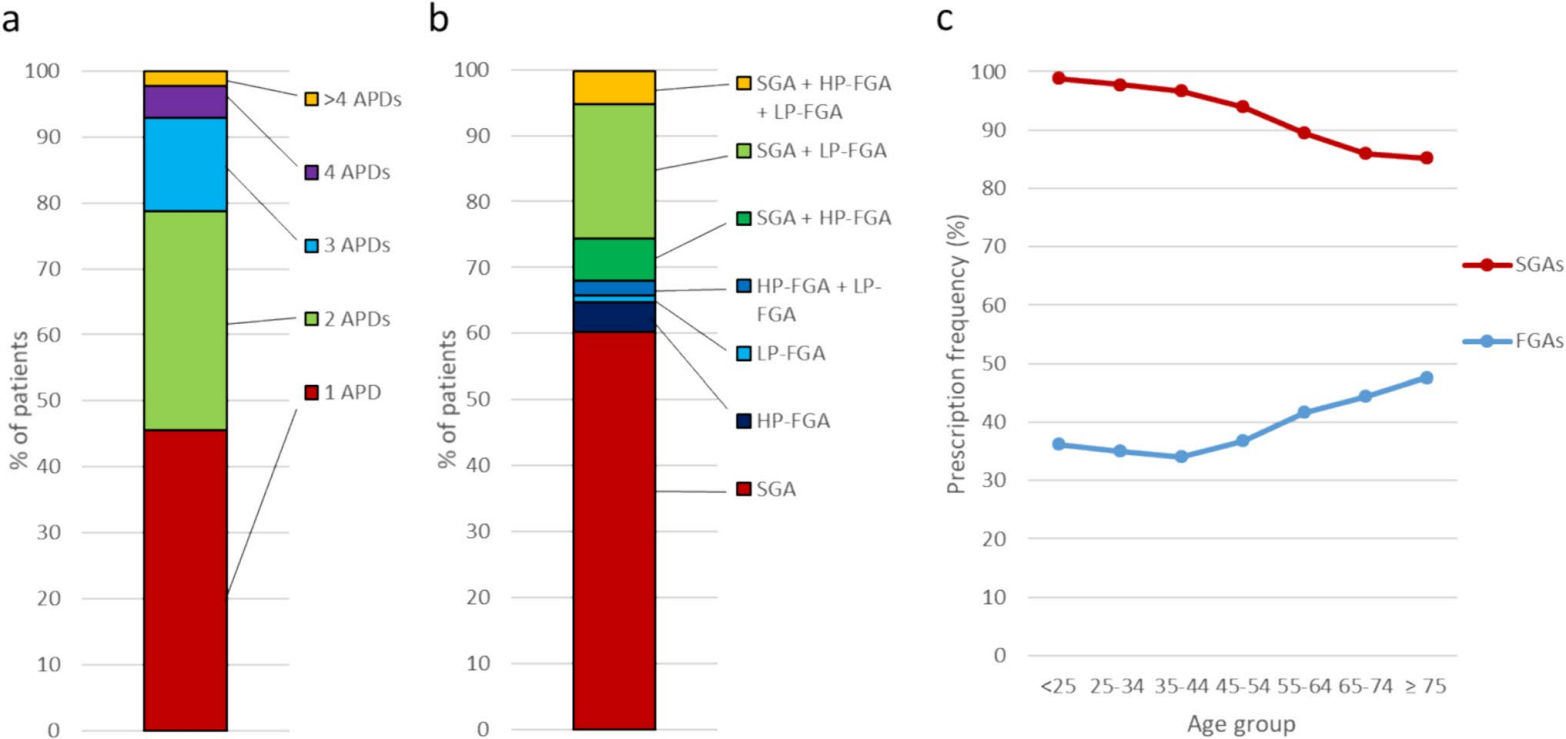
Prescription frequencies. **a** Prescription frequencies according to the number of antipsychotics prescribed to patients. **b** Prescription frequencies by the class of the antipsychotic drug. **c** Prescription frequencies by age group, shown separately for first-generation and second-generation antipsychotics. HP-FGA, high-potency first-generation antipsychotic drugs; LP-FGA, low-potency first-generation antipsychotic drugs; SGA, second-generation antipsychotic drugs; FGA, first-generation antipsychotic drugs

According to the inclusion criteria, all patients used at least one APD during the observation period. Most patients (92.0%) had at least one SGA as sole or combination therapy, and 17.0% of patients had at least one high-potency FGA alone or in combination with an SGA or a low-potency FGA. During the observation period, 45.6% of all patients were prescribed only one specific antipsychotic, 33.1% were prescribed two different APDs, 14.3% received prescriptions for three different APDs, and 7% received four or more different APDs (Fig. 1a).

Figure 1 displays the prescription frequencies of APDs categorized into low-potency and high-potency FGAs and SGAs and their combinations (1b), and prescription frequencies of FGAs and SGAs by age group (1c). Prescription of SGAs was the most common overall. Sixty percent of patients were prescribed SGAs alone without additional FGAs, and 92% of patients were prescribed SGAs either alone or in combination with high- or low-potency FGAs, with age-related differences, as shown in Fig. 1c. In the youngest age group under 25 years, almost all patients (98.8%) were prescribed SGAs alone or in combination with FGAs. With increasing age, the prescription frequency of SGAs continuously decreased and was 85.2% in the oldest age group (≥ 75 years). Conversely, the prescription frequency of FGAs increased from 36.2%, 35.0%, and 34.1% in the < 25 years, 25–34 years, and 35–44 years age groups, respectively, to 44.4% and 47.6% in patients aged 65–74 years and ≥ 75 years, respectively.

**Fig. 2:**
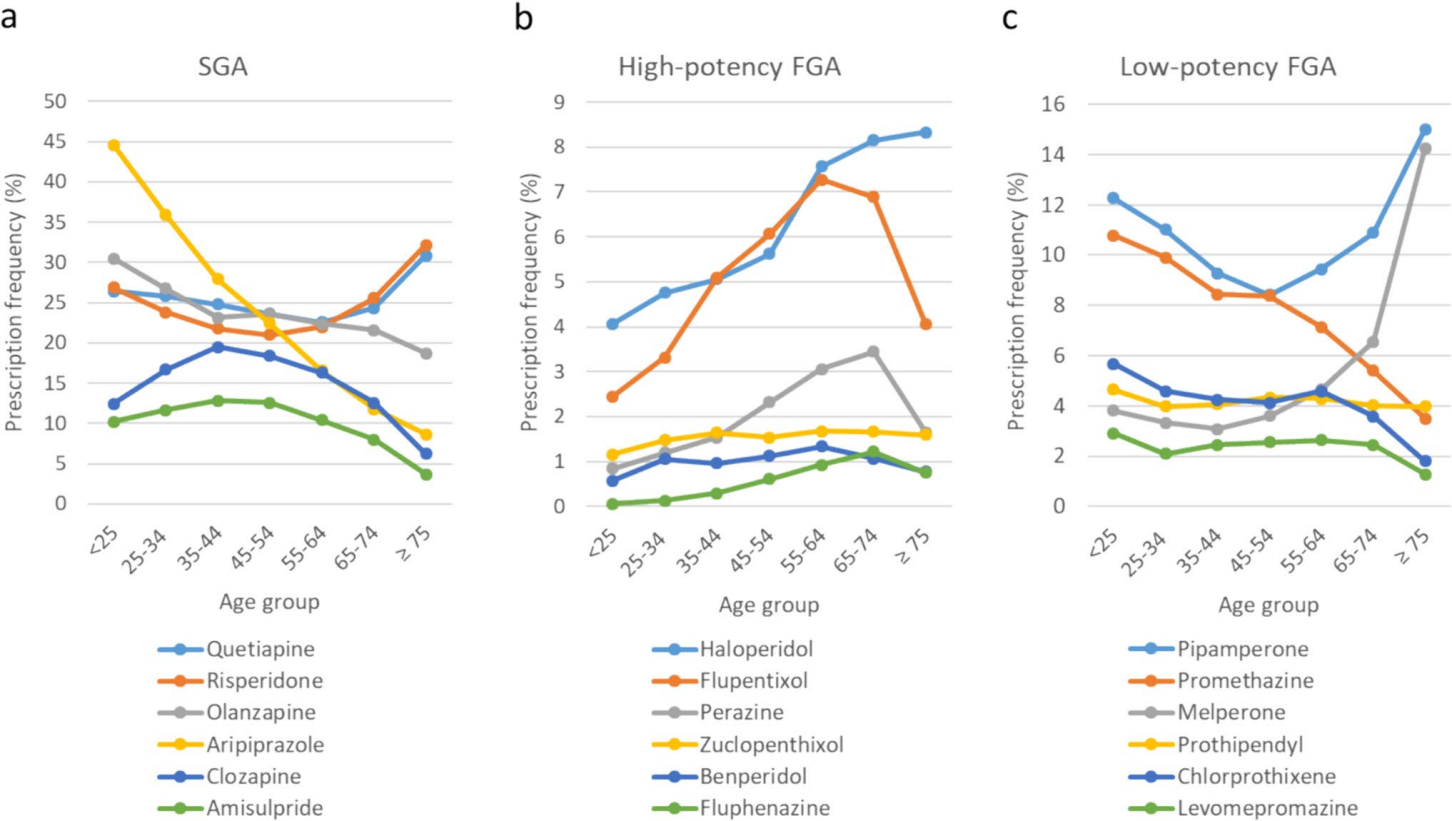
Prescription frequencies of individual antipsychotics by age group. Figures **a**–**c** show the prescription frequencies of second-generation antipsychotics (SGA) and high-potency and low-potency first-generation antipsychotics (FGA). It should be noted that the overall prescription frequency, i.e., the sum of the prescription frequencies of all antipsychotic drugs, within an age group can exceed 100% due to the concomitant prescription of different drugs

Figure 2 shows the prescription frequencies of individual APDs by age. Among the SGAs, the prescription frequencies of aripiprazole and olanzapine peaked in the under-25 age group (44.6% and 30.5%, respectively), while the prescription frequencies of clozapine and amisulpride peaked in the 35–44 years age group (19.5% and 12.8%, respectively). Risperidone and quetiapine were prescribed most frequently in the over-75 age group (32.2% and 30.9%, respectively). In the geriatric age group over 65 years, the prescription frequencies of risperidone, quetiapine and olanzapine together accounted for the largest share, while all other SGAs played only a minor role.

The high-potency FGAs were generally prescribed infrequently with a consistent age-dependent increase in prescription frequencies up to the 55- to under-65 age groups. With the exception of haloperidol, which was by far the most frequently prescribed high-potency FGA in the over-75 s (prescription frequency 8.3%), the prescription frequencies of the high-potency FGAs declined in geriatric patients, in some cases markedly.

For the low-potency FGAs, the prescription frequencies in geriatric patients decreased, with the exception of pipamperone and melperone, which were by far the most frequently prescribed in patients over 75 years of age (prescription frequencies 15.0% and 14.3%, respectively). Promethazine, the second most frequently prescribed low-potency FGA in the under-25 s after pipamperone, is hardly prescribed any more in the over-75 s.

**Fig. 3:**
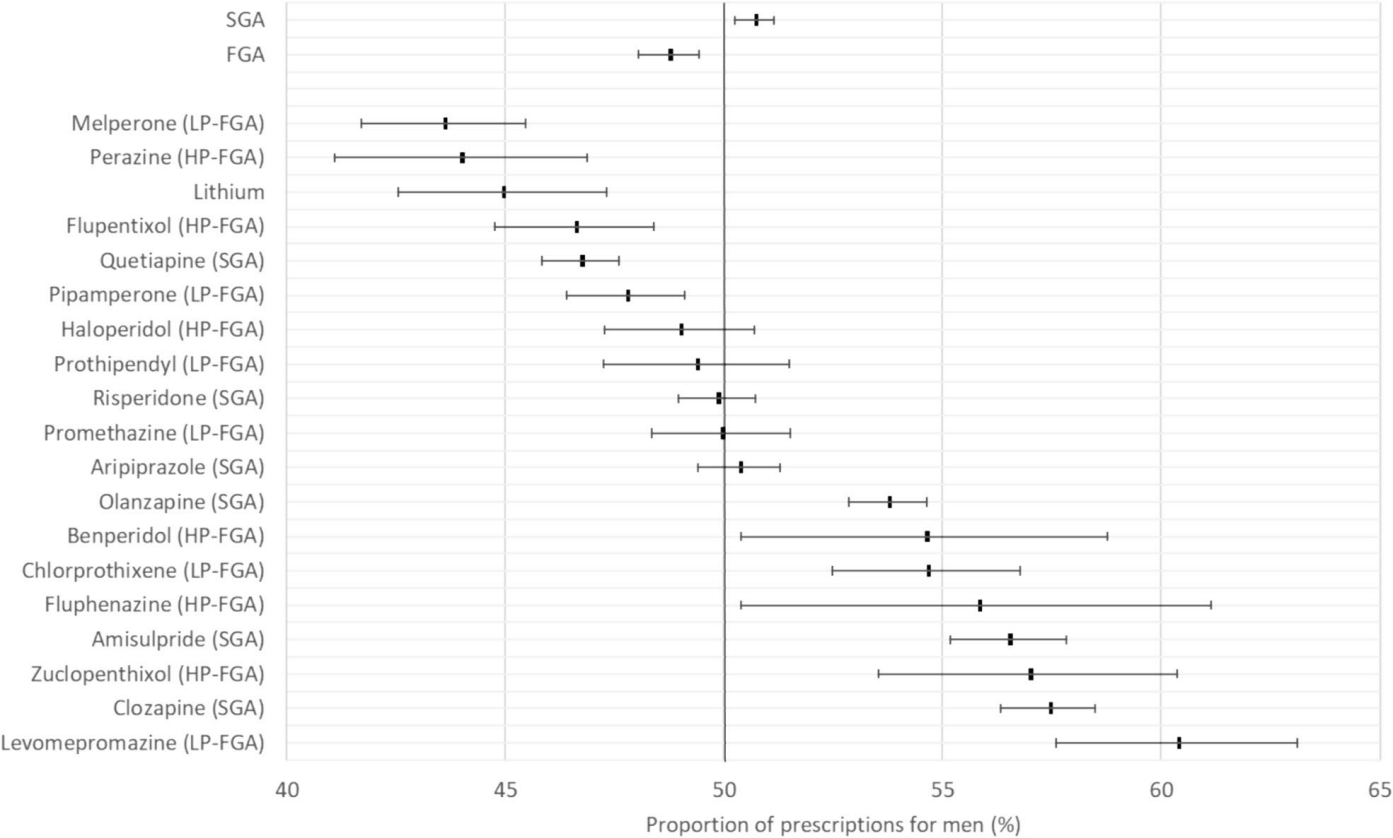
The relative proportion of prescriptions for the specified drug or drug class for male patients, in comparison to the total number of prescriptions for both male and female patients, is provided. This proportion is expressed as a percentage with the 95% confidence interval. FGA, first-generation antipsychotic drug; SGA, second-generation antipsychotic drug; HP-FGA, high-potency first-generation antipsychotic drug; LP-FGA, low-potency first-generation antipsychotic drug

When comparing the prescription frequencies of men and women, essentially balanced sex ratios were found for the two drug classes of FGAs and SGAs, as shown in Fig. 3. However, for some drugs, there were significant differences in prescription frequencies between men and women, e.g., 1.5-fold and 1.3-fold higher prescription frequencies of levomepromazine and clozapine, respectively, in men or a 1.3-fold higher prescription frequency of melperone in women.

### Treatment intensity of APDs

**Fig. 4:**
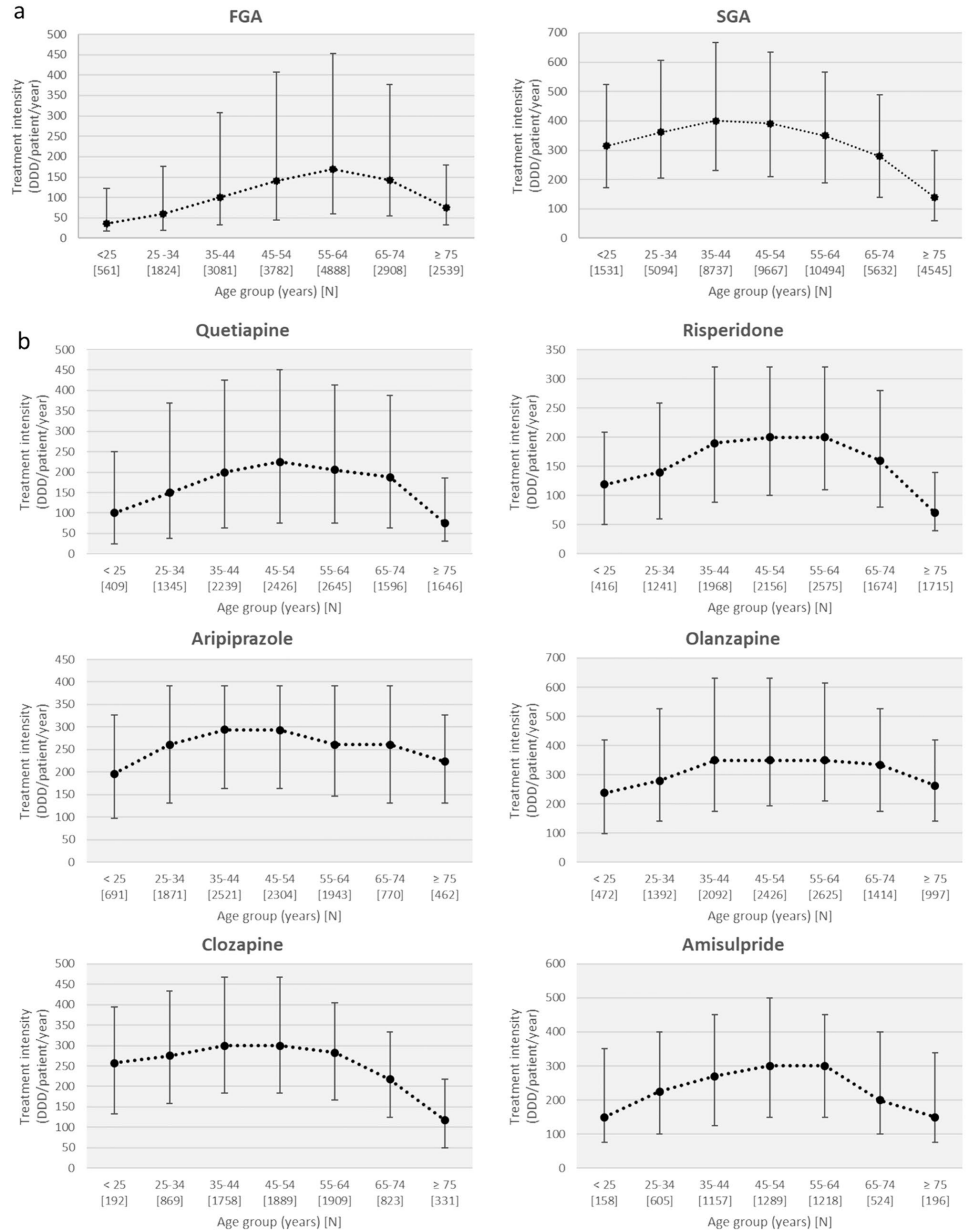

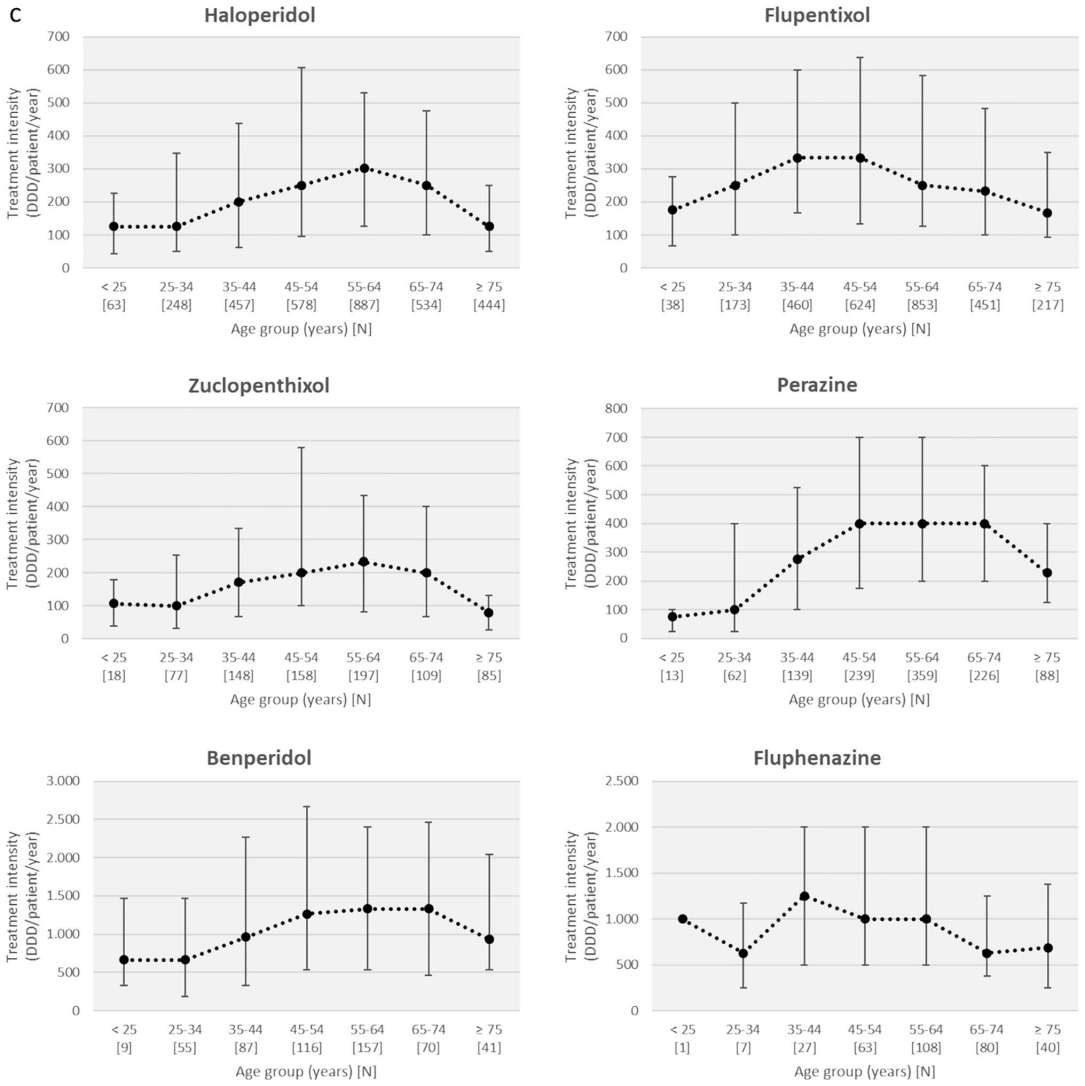

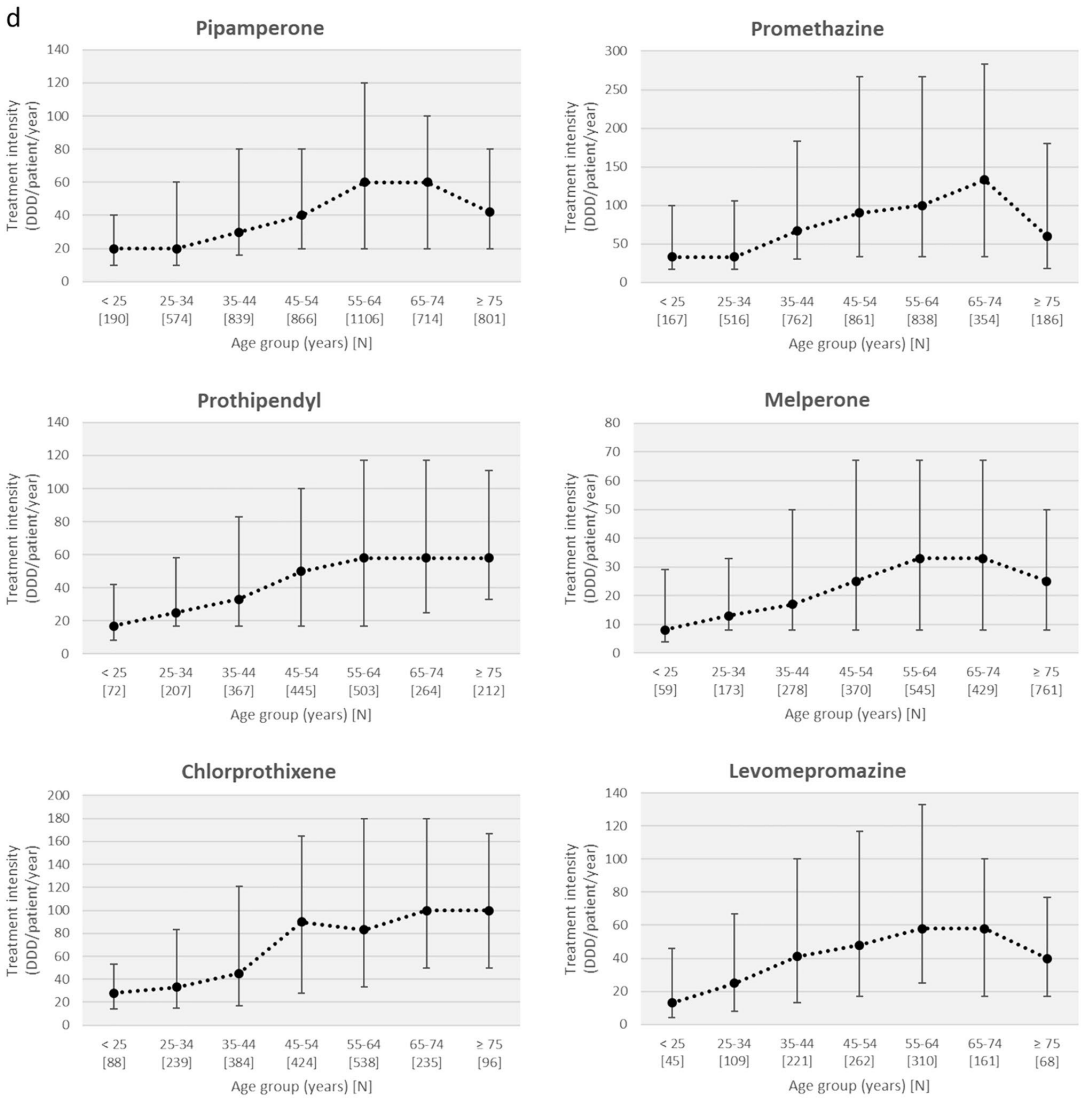
Median values with the interquartile range of treatment intensities (defined daily dose [DDD]/patient/year) for first-generation (FGA) and second-generation (SGA) antipsychotics (**a**) and for individual antipsychotic drugs **(b**–**d)**, stratified by patient age group

As a rough estimate of APD consumption, we calculated the treatment intensities of APDs. Figure 4 shows the median values with the interquartile range of treatment intensities overall for FGAs and SGAs or for each APD individually, stratified by patient age group. Basically, we observed a bell-shaped relationship between patient age and treatment intensity. Treatment intensity with SGAs initially increased with age, peaked among 35- to 44-year-olds, and then decreased, with the lowest treatment intensity in patients ≥ 65 years of age. Treatment intensity with FGAs peaked among those aged 55 to 64 years and then decreased in geriatric age groups, but less sharply compared with SGAs.

Figures 4b–d show treatment intensities by age for individual APDs. With few exceptions, the bell-shaped relationships between patient age and treatment intensities are also evident for most individual APDs. For several drugs, treatment intensity, expressed as median DDD per patient per year, was markedly lower in geriatric patients than in middle-aged patients. For example, treatment intensity of haloperidol, zuclopenthixol, quetiapine, risperidone, and clozapine decreased by 59%, 66%, 64%, 65%, and 59%, respectively, in patients older than 75 years compared with the 55- to 64-year-old group (Table S2).

**Fig. 5:**
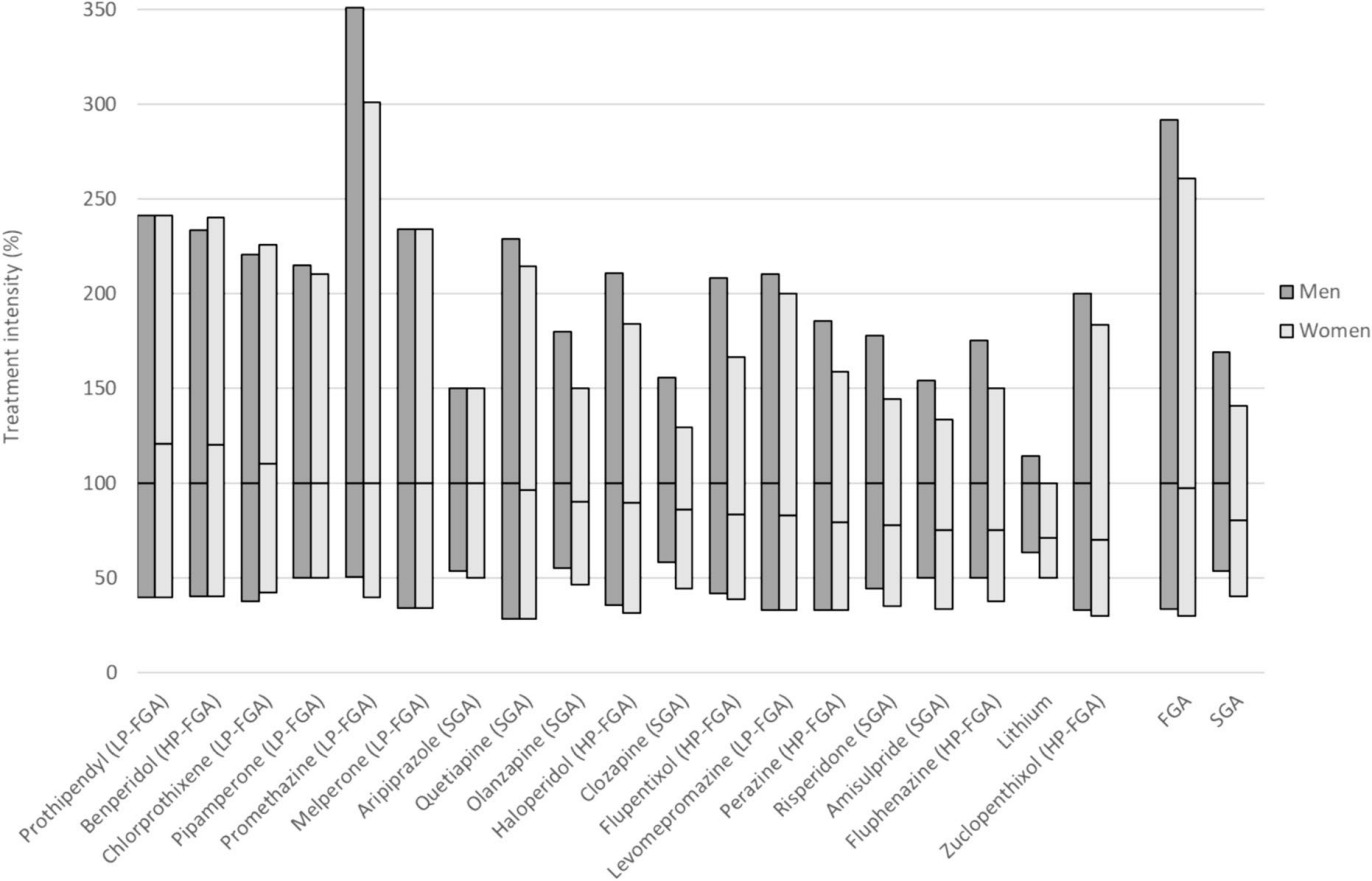
Relative treatment intensity for the specified drugs and drug classes for women, normalized to the corresponding treatment intensity for men (set to 100%). The boxes show the median values with the interquartile range. For none of the drugs treatment intensities were significantly different between men and women. FGA, first-generation antipsychotic drug; SGA, second-generation antipsychotic drug; HP-FGA, high-potency first-generation antipsychotic drug; LP-FGA, low-potency first-generation antipsychotic drug

Figure 5 shows differences of APD exposure by sex. Sex differences in treatment intensity, with reductions of up to approximately 25% in women versus men, were observed for some but not all antipsychotics.

**Fig. 6:**
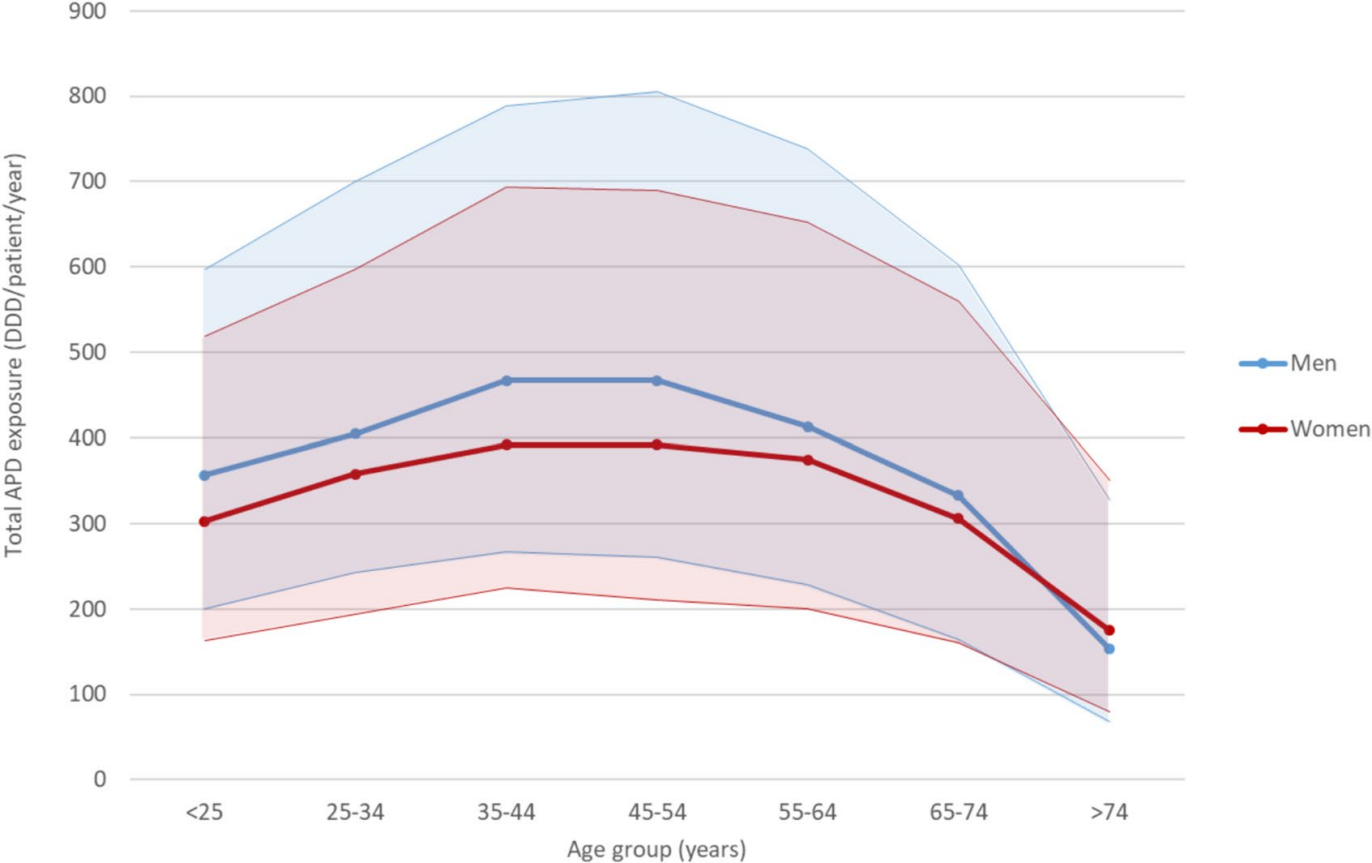
Total exposure to antipsychotic drugs (ATC groups N05A, excluding N05AN01 [lithium], including N05CM22 [promethazine]) as the sum of all antipsychotic defined daily dose [DDD] equivalent per patient and year, stratified by sex and age group. Median with interquartile range

Many patients take multiple APDs in combination, each contributing to the total APD exposure. Therefore, we also analyzed the total exposure to all APDs dispensed to each patient during the study period, stratified by age. Total APD exposure first increased slowly with age, then peaked among 35- to 54-year-olds, and then declined, with the steepest decline among those aged 75 and older compared with the 65–74 age group. Total APD exposure was generally higher in men than in women. However, the sex difference decreased with age and was no longer present in those over 75 years of age (Fig. 6).

### Co-medication

**Fig. 7:**
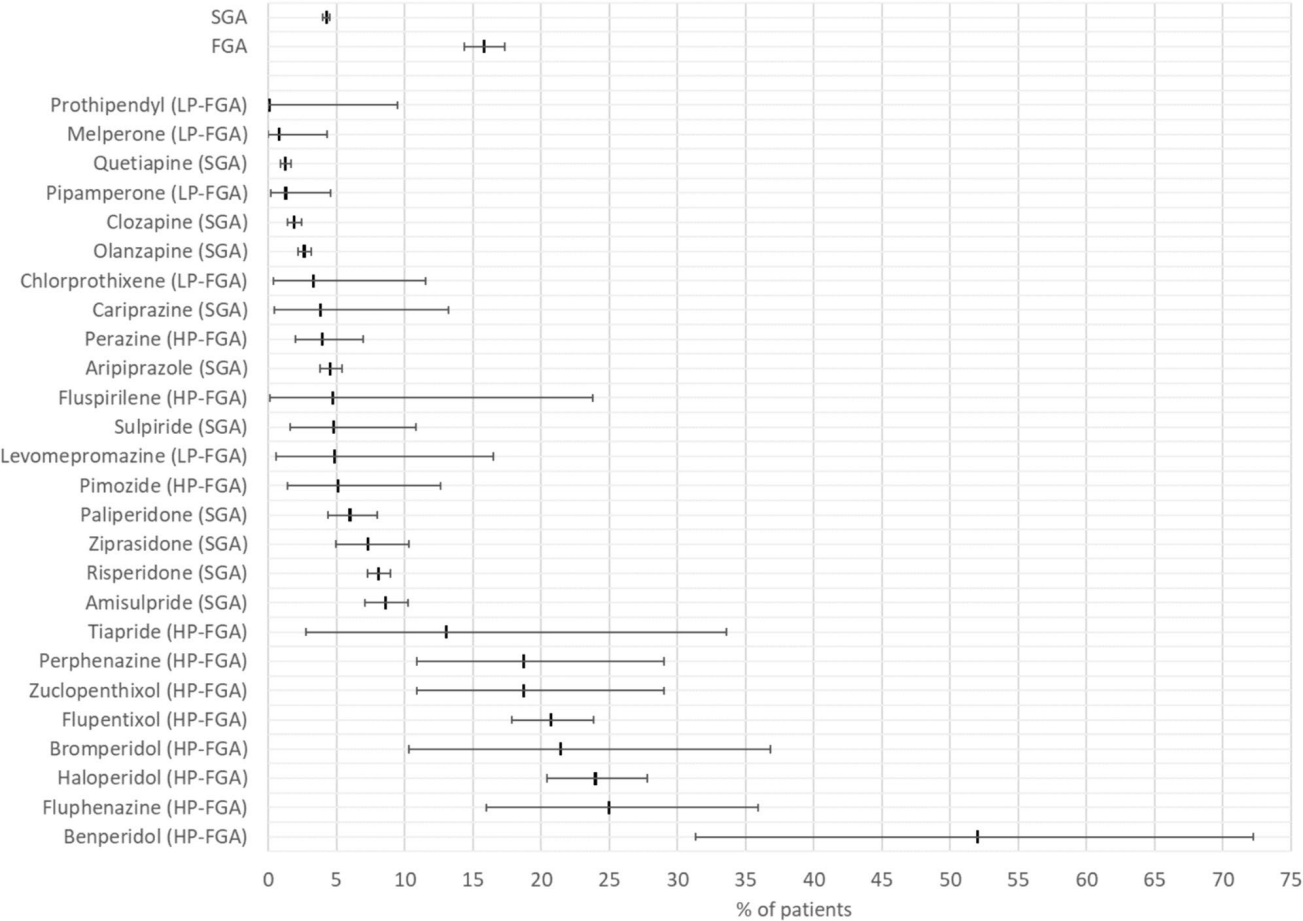
Prescription frequencies with 95 percent confidence interval of biperiden in patients prescribed the indicated antipsychotic drug as monotherapy, i.e., without prescription of another antipsychotic. *FGA* first-generation antipsychotic drug; *SGA* second-generation antipsychotic drug; *HP-FGA* high-potency first-generation antipsychotic drug; *LP-FGA* low-potency first-generation antipsychotic drug

Anxiolytics (prescription frequency 18.9%), antidepressants (32.0%), analgesics (20.2%), antiparkinsonian agents (12.4%, mostly biperidene), lithium salts (3.3%), and anticonvulsants (14.9%, mostly valproate) were frequently prescribed as concomitant medications acting in the central nervous system (Table S1). The most frequently prescribed drugs were the analgesic metamizole (prescription frequency 17.2%), the anxiolytic lorazepam (13.9%), the anticholinergic biperiden (10.1%), the anti-epileptic valproic acid (6.4%) and the five antidepressants sertraline (6.6%), mirtazapine (5.2%), venlafaxine (4.6%), citalopram (4.5%), and escitalopram (3.1%). Biperiden, the third most common co-medication, is a muscarinic receptor antagonist used to treat parkinsonism and to control extrapyramidal side effects of APDs, such as dystonia, tardive and tardive dyskinesia. Figure 7 shows the prescription frequencies of biperiden in patients prescribed an APD as monotherapy. Biperiden was prescribed more frequently together with FGAs (prescription frequency 15.8%) than with SGAs (4.3%). Biperiden prescription frequencies were particularly high in patients with the high-potency FGAs benperidol (52.0%), fluphenazine (25.0%), haloperidol (24.0%), bromperidol (21.4%), flupentixol (20.7%), perphenazine (18.8%), and zuclopenthixol (18.8%). In contrast, biperiden prescription frequencies were least frequent in patients with the SGAs quetiapine (1.2%), clozapine (1.9%), and olanzapine (2.6%) and the low-potency FGAs melperone (0.8%) and pipamperone (1.3%).

## Discussion

The primary objective of our study was to explore the choice of APDs and treatment intensity according to the age of schizophrenia patients within a naturalistic outpatient setting, utilizing health claims data. Our focus on the outpatient setting is crucial, given its significant divergence from the inpatient setting in terms of patient characteristics and treatment approaches. In inpatient settings, patients often exhibit more severe symptoms and receive close monitoring, allowing for more intensive and immediate medication adjustments. Conversely, outpatient treatment focuses on maintaining remission, preventing relapses, improving quality of life, minimizing symptoms and functional impairments, reducing side effects, and promoting recovery. These goals can influence the selection and intensity of antipsychotic treatment in the outpatient setting.

Our findings indicate a preference for SGAs across all age groups, with particularly high usage among younger patients. This preference may be attributed to clinical guidelines favoring SGAs due to their improved side effect profile compared to FGAs [3]. SGAs are linked to fewer extrapyramidal side effects, including tardive dyskinesia, which can be especially problematic in elderly patients. Additionally, SGAs may be perceived as more effective in addressing both positive and negative symptoms of schizophrenia, possibly explaining their increased use in younger patients [26]. However, it is noteworthy that in older age groups, prescription frequencies for SGAs are increasing only for specific drugs such as quetiapine and risperidone, while decreasing for all other drugs. This shift signifies a fundamental change in prescribing practices for SGAs in older individuals.

The scarcity of comprehensive evidence regarding the efficacy and safety of antipsychotics in older individuals with schizophrenia poses a significant challenge. Clinical trials often exclude this population, impeding the establishment of precise treatment guidelines. Although the German S3 guideline for schizophrenia dedicates a section to treatment in older age, it refrains from making specific recommendations for or against the use of certain drugs in older schizophrenia patients due to insufficient evidence from clinical trials [3]. Nevertheless, the guideline references a survey of American experts on antipsychotic use in elderly patients, recommending risperidone (at a dose of 1.25–3.5 mg/day) as first-line treatment and quetiapine (100–300 mg/day), olanzapine (7.5–15 mg/day), and aripiprazole (15–30 mg/day) as second-line treatment for late-life schizophrenia [27]. The updated PRISCUS list (Priscus 2.0), a catalog of substances potentially unsuitable for the elderly and thus to be avoided, designates commonly prescribed SGAs, including risperidone and quetiapine, as potentially inappropriate medications (PIMs) if used for more than 6 weeks [28]. However, the PRISCUS list lacks differentiation between indications for which antipsychotics can be prescribed in older individuals, such as the treatment of agitated dementia, geriatric psychotic major depression, or late-life schizophrenia [28]. The American Geriatrics Society's 2023 updated AGS Beers criteria emphasize avoiding antipsychotics—both FGAs and SGAs—except for FDA-approved indications like schizophrenia [29].

Our study also identified variations in prescribing frequencies of low-potency FGAs among different age groups. While these drugs were generally less frequently used in older age groups, the use of pipamperone and melperone increased significantly. This trend may be attributed to the reduced anticholinergic effects of butyrophenones like the low-potency FGAs pipamperone and melperone. Moreover, these medications are well-suited for managing psychomotor agitation and inducing sleep, particularly in the elderly population [30]. Additionally, they exhibit minimal proconvulsive effects [31]. These favorable characteristics, distinct from other low-potency FGAs, are reflected in the PRISCUS list, which suggests both substances as potential therapeutic alternatives for potentially inappropriate APDs in older patients [28].

High-potency FGAs showed varied prescription patterns, with usage generally increasing with age. However, flupentixol and perazine saw a significant decline in the oldest age groups. In contrast, haloperidol prescriptions increased, despite a decline in treatment intensity from age 65, as illustrated in Fig. 4c. The reason for the increasing prescriptions of haloperidol with age is unclear. Patients with early-onset schizophrenia may continue haloperidol treatment initiated before most SGAs were introduced in the 1990s. However, there is no clinical evidence supporting haloperidol's superiority in the chronic outpatient treatment of elderly schizophrenia patients. SGAs significantly outperformed FGAs, including haloperidol, in terms of negative symptoms, global cognitive function, and relapse rate [32]. While haloperidol is effective for managing positive symptoms, its use is limited by side effects like extrapyramidal symptoms and increased risk of QT prolongation. Additionally, comorbidities common in older patients, such as Parkinson's disease or heart disease, contraindicate haloperidol or require careful use. Therefore, the increasing prescription frequency with age, where more than 8% of patients over 65 are prescribed haloperidol, is surprising.

Our study revealed sex differences in prescription frequencies and treatment intensities. A recent literature review on gender differences in antipsychotic response concluded that women’s psychotic symptoms generally respond at lower doses than those of men [13]. However, according to the results of the review article, the effective doses after menopause are similar in men and women [13]. The review’s conclusions are consistent with the results of our study: total APD exposure was lower in women of childbearing age than in men, with the greatest difference in the 35–54 age group. This difference gradually disappeared with age beyond 55 years.

Despite some disagreement among clinical data, female sex was more often associated with greater weight gain in patients on APs in clinical studies [33]. Iverson et al. reported that twice as many females described the side effect burden of APDs as severe [14]. Kraal et al. found that females on clozapine or olanzapine appear to represent a high-risk group for metabolic dysfunction [34]. Barbui et al. provided evidence that women tend to report antipsychotic drug side effects, including weight gain, anticholinergic reactions, and extrapyramidal side effects, more frequently than men. Sex, after adjusting for possible confounders, was the strongest determinant of the subjective tolerability of antipsychotic drugs [35]. Sex differences in the frequency of side effects may affect adherence to APDs and could explain lower prescription frequencies in females compared to males, particularly for antipsychotics strongly associated with metabolic dysfunction such as clozapine and olanzapine. Sex-dependent differences in treatment intensity for individual APDs may result from variations in target symptoms, pharmacokinetics, and tolerability between sexes. Data on the pharmacokinetics and pharmacodynamics of APDs by sex have scarcely been systematically investigated, leading to a lack of sex-specific dosage recommendations in SmPCs and guidelines.

One noteworthy aspect of our findings is the concurrent prescription of biperiden alongside antipsychotic medications, particularly among patients receiving an FGA. Biperiden, an anticholinergic medication, is frequently prescribed to manage extrapyramidal symptoms associated with antipsychotic treatment, such as dystonia and early or tardive dyskinesia [36]. Our data indicate that biperiden is more commonly co-prescribed with FGAs compared to SGAs, with specific high-potency FGAs such as benperidol, fluphenazine, and haloperidol showing a higher likelihood of co-prescription with biperiden. This prescribing pattern aligns with the traditional use of anticholinergics to alleviate the side effects commonly associated with FGAs. However, as somatic diagnoses were not available for our analysis, we cannot exclude with certainty that in some cases biperiden may have been prescribed primarily to treat Parkinson's disease rather than an APD-induced movement disorder. Nevertheless, it is important to note that while biperiden can effectively address acute movement-related side effects, its long-term use has adverse effects, including cognitive impairment and anticholinergic burden, which are of particular concern in older patients [37]. The decision to co-prescribe biperiden should thus be carefully considered, and clinicians should weigh the benefits against the potential risks, particularly in elderly individuals who may be more vulnerable to adverse effects. The surprisingly frequent prescription of biperiden contradicts the German guideline for treating schizophrenia [3], which discourages the long-term use of anticholinergics like biperiden for managing extrapyramidal motor side effects such as tardive dyskinesia.

### Strength and limitations

This study has several strengths. It uses the GePaRD database, encompassing health insurance data from about 20% of the German population, providing a robust and representative dataset for analysis. The study offers a nuanced understanding of APD prescribing by examining age groups by decade and considering sex differences. It distinguishes between FGAs and SGAs and has sufficient statistical power to analyze individual APDs. Our study allows us to identify discrepancies between age- and sex-dependent antipsychotic prescribing practices for schizophrenia and available clinical evidence and guideline recommendations.

Despite these strengths, the study has limitations. It focuses on prescription patterns and treatment intensities of APDs without examining clinical outcomes or patient responses. It includes only outpatient APD prescriptions, missing insights from inpatient settings where FGAs are often prescribed, limiting a comprehensive view of schizophrenia treatment with APDs in Germany. Reliance on data from the German healthcare system may affect the generalizability of the results to other countries. The database did not provide detailed data on the severity of schizophrenia symptoms or the presence and severity of somatic comorbidities, which may influence treatment decisions, so these factors were not incorporated into the analysis. Additionally, patients were not always followed for a full year (e.g., if they joined the health insurance after the start of the observation year or left it before the end of the year for various reasons, including death), potentially biasing the estimation of treatment intensity.

## Conclusion

The study's major findings include a notable preference for SGAs across all age groups, especially in younger patients, possibly due to their perceived better tolerability and efficacy. The prescription patterns of specific SGAs and FGAs varied across age groups, highlighting the complexity of treatment decisions in schizophrenia management. Sex differences in prescribing frequencies and treatment intensities were also observed, further highlighting the need for personalized treatment approaches. The need for sex-dependent adjustments to APD treatment may arise from differences in disease presentation, treatment response, and the higher risk of adverse effects in female patients compared to males [13]. The physiological causes can be attributed to hormonal differences. Hormonal status, particularly estrogen levels, affects the pharmacokinetics of APDs by influencing the expression and activity of drug-metabolizing enzymes. Lower estrogen levels after menopause may explain the convergence in APD dosage between men and women in older age groups, as observed in our study [38]. Furthermore, the study provides data on the frequent concomitant prescription of biperiden, which is most likely used to treat the adverse extrapyramidal effects of APDs. This prescribing practice exposes elderly schizophrenia patients to an increased anticholinergic burden without sufficient evidence of efficacy in the treatment of antipsychotic-induced tardive dyskinesia [36].

While this research offers valuable insights into APD utilization, it underscores the importance of future studies that incorporate clinical context and explore the reasons behind prescription choices. The treatment of schizophrenia in older adults remains a complex and evolving field, necessitating a deeper understanding of real-world practices to optimize care for this vulnerable population.

## Supplementary Information

Below is the link to the electronic supplementary material.

Supplementary file1 (DOCX 15 KB)

## Data Availability

As we are not the owners of the data we are not legally entitled to grant access to the data of the German Pharmacoepidemiological Research Database. In accordance with German data protection regulations, access to the data is granted only to employees of the Leibniz Institute for Prevention Research and Epidemiology – BIPS on the BIPS premises and in the context of approved research projects. Third parties may only access the data in cooperation with BIPS and after signing an agreement for guest researchers at BIPS.
