# Supplementary material for: Age and sex differences in outpatient antipsychotic prescriptions for schizophrenia: a claims data study
Source: Eur Arch Psychiatry Clin Neurosci. 2024 Sep 30;275(5):1403–17. doi: 10.1007/s00406-024-01867-z (PMC12271300; doi:10.1007/s00406-024-01867-z)
Supplement: Supplementary file 1 — Supplementary file1 (DOCX 15 KB) [file 406_2024_1867_MOESM1_ESM.docx]

**Supplementary Material**

**to**

**Age and sex differences in outpatient antipsychotic prescriptions for schizophrenia: A claims data study**

**by**

**T. Ramin, J.-U. Peter, M. Schneider, M. Heinze, O. Riedel, S. H. Langbein, U. Haug, O. Zolk**

**Table S1** Prescription frequencies by ATC drug class

| **Drug class** | **ATC Code** | **Subclass** | **Prescription frequency %** | **Patients**  **N** |
| --- | --- | --- | --- | --- |
| Psycholeptics | N05 |  | 100.0 | 49,681 |
| Antipsychotic drugs | N05A | First generation | 39.4 | 19,583 |
|  |  | Second generation | 92.0 | 45,700 |
| Anxiolytics | N05B |  | 18.9 | 9,376 |
| Lithium salts | N05AN |  | 3.3 | 1,654 |
| Psychoanaleptics | N06 |  | 33.0 | 16,385 |
| Antidepressants | N06A |  | 32.0 | 15,883 |
| Analgesics | N02 |  | 20.2 | 10,043 |
| Antiepileptics | N03 |  | 14.9 | 7,417 |
| Antiparkinson drugs | N04 |  | 12.4 | 6,138 |
| Other nervous system drugs | N07 |  | 1.4 | 671 |

**Table S2** Treatment intensity of antipsychotics in older patients compared to patients aged 55-64 years. The treatment intensity, i.e., the median of the defined daily dose/patient/year in patients aged 55 to 64 years was set at 100%. IQR, inter-quartile range

|  | **Age group 65-74 years** | | **Age group ≥75 years** | |
| --- | --- | --- | --- | --- |
|  | **Relative treatment intensity in % (IQR)** | **N** | **Relative treatment intensity in % (IQR)** | **N** |
| First-generation antipsychotic drugs | |  |  |  |
| Flupentixol | 93 (40;193) | 451 | 67 (37;140) | 217 |
| Haloperidol | 83 (33;157) | 534 | 41 (17;83) | 444 |
| Zuclopenthixol | 86 (28;171) | 109 | 34 (11;57) | 85 |
| Perazine | 100 (50;150) | 226 | 57 (31;100) | 88 |
| Benperidol | 100 (35;185) | 70 | 70 (40;153) | 41 |
| Fluphenazine | 83 (33;157) | 80 | 69 (25;138) | 40 |
| Pipamperone | 100 (33;167) | 714 | 70 (33;133) | 801 |
| Promethazine | 134 (34;284) | 354 | 61 (18;180) | 186 |
| Melperone | 100 (25;199) | 429 | 75 (25;149) | 761 |
| Prothipendyl | 100 (43;199) | 264 | 100 (57;190) | 212 |
| Chlorprothixene | 120 (60;216) | 235 | 120 (60;199) | 96 |
| Levomepromazine | 100 (28;171) | 161 | 68 (28;132) | 68 |
| Second-generation antipsychotic drugs | |  |  |  |
| Quetiapine | 91 (30;188) | 1596 | 36 (15;90) | 1646 |
| Risperidone | 80 (40;140) | 1674 | 35 (20;70) | 1715 |
| Olanzapine | 96 (50;150) | 1414 | 75 (40;120) | 997 |
| Aripiprazole | 100 (50;150) | 770 | 85 (50;125) | 462 |
| Clozapine | 76 (44;118) | 823 | 41 (18;76) | 331 |
| Amisulpride | 67 (33;133) | 524 | 50 (25;113) | 196 |

The median DDD in patients 55-64 years old was set at 100%.
